# An office-based fix-and-follow grading system assessing visual function in preverbal children

**DOI:** 10.1186/s12886-021-02187-9

**Published:** 2021-11-30

**Authors:** Hyeshin Jeon, Jae Ho Jung, Hee-young Choi

**Affiliations:** 1 Department of Ophthalmology, School of Medicine, Pusan National University, 1-10 Ami-dong Seo-gu, Busan, 49241 South Korea; 2 Biomedical Research Institute, Pusan National University Hospital, Busan, South Korea; 3 Department of Ophthalmology, Seoul National University College of Medicine and Seoul National University Hospital, Seoul, South Korea

**Keywords:** Fix-and-follow, Saccade, Visual development, Visual screening

## Abstract

**Background:**

Assessing visual function in infants is usually challenging. Fix-and-Follow is a simple and popular method for assessing early development of visual perception in infants, currently however, there is no formal reproducible method for grading the capacity of fix-and-follow. This study was to develop and validate a new fix-and-follow grading system for assessing visual function development in preverbal children.

**Methods:**

In this cross-sectional study, the fix-and-follow grades was evaluated in 21 consecutive preverbal children. Fixation was categorised as grade 1 if there was no response to the target and grade 2 if there was a response but only for < 3 s. Grades of 3 and 4 were assigned based capacities to (1) fix on a moving target for ≥3 s, and (2) shift fixation from one target to another. If only one of these two criteria was met, grade 3 was assigned. If both were met, grade 4 was assigned. Following was evaluated using smooth pursuit movement, where grade 1 indicated no movement, grade 2 partial movement, and grade 3 complete movement. Two ophthalmologists independently applied the grading method in all patients. Then one of two examiners repeated the examinations to investigate the intra-observer agreement of the grading system.

**Results:**

Intra-observer agreement was excellent (Kappa coefficient = 0.823) and inter-observer agreement was good (Kappa coefficient = 0.625). All patients who exhibited abnormal ocular movement had score discrepancy between a new fix-and-following grading examination.

**Conclusions:**

The new fix-and-follow grading scale can be applied easily in preverbal children in an office setting, and it proved reliable and reproducible.

## Background

Both visual sensory and oculomotor functions are essential for the normal development of visual perception [1]. Examination of sensory visual function including visual acuity, visual field and color vision in infants is usually challenging due to the difficulty of ensuring cooperation, and their relatively undeveloped capacity for verbal expression. Alternative methods including visual evoked cortical potential (VEP) and the forced choice preferential looking test (FPL) have been used, [2] but these tests are usually not available as a part of routine paediatric eye examination due to a lack of equipment, time or experience. Conversely, the evaluation of oculomotor responses to visual stimulation can be performed easily by observing the behaviour of the subject, even in cases where the subject is relatively uncooperative.

The oculomotor functions associated with visual perception include accommodation, vergence, saccades, smooth pursuit and optokinetic nystagmus. Of them, whether the subject can orient the fovea to fix on a target and maintain fixation of a moving target is referred to as ‘fix-and-follow’. Most neonates can already fix-and-follow to some extent immediately after birth [3]. The ability to fix on a target and follow it is one of the principal tests that can be used as an indicator of early development of visual perception in infants, and it is a simple and popular method used by general and paediatric ophthalmologists [4]. Currently however, there is no formal reproducible method for quantitative measurement of the capacity of children to fix-and-follow. The purpose of the current study was to test the feasibility of a new office-based fix-and-follow grading system in preverbal children.

## Methods

The Institutional Review Board of Pusan National University Hospital approved this study. All medical procedures were performed in accordance with the tenets of the Declaration of Helsinki. Because it is a retrospective study and the evaluation items were included in the routine ophthalmic examination, informed consent was not obtained. Preverbal infants who attended for ophthalmic evaluation were included in the study. We investigated the demographic characteristics of the patients including age, sex, birth history and the presence of underlying systemic disease or developmental delay. Full ophthalmic assessments including fundus examination, slit-lamp anterior segment examination, cycloplegic refraction, ocular deviation and evaluation of eye movement were performed.

### Fix-and-follow grading system

Fix-and-follow was evaluated at a distance of 30 cm under binocular conditions using target pictures of animals that were 1 cm in diameter, with keeping the patient’s head still. The fix-and-follow grading system is described in Table 1. The grading score was defined as the sum of fix-and-follow grade ranges from 2 (worst possible score) to 7 (best possible score).

**Table 1:** Components of the fix-and-follow grading system

**Fixation:** The target for fixation was located at a distance of 30 cm, and the test was performed under binocular conditions	
Level 1	No response to the target
Level 2	A response to the target, but only for < 3 s
Level 3	Only one of the two criteria below (1 and 2) were met
Level 4	Both of the two criteria below were met
(1) Fixed on the target for ≥3 s	
(2) Saccadic eye movement from one target to another	
**Following:** Horizontal smooth pursuit eye movement within 30-degree ranges	
Level 1	No movement
Level 2	Partial movement
Level 3	Complete movement

To evaluate the inter-observer reproducibility of the fix-and-follow grading system, two well-trained ophthalmologists (HJ and HC) independently applied the grading method in all patients. HJ then repeated the examinations to investigate the intra-observer agreement of the grading system. To reduce bias, there was a 4–6 weeks interval between the initial measurement and repeated measurement in all patients.

### Data analysis

We included 21 subjects, which determined according to clinical situation, given the lack of certainty regarding the benefit of the proposed technique.

Cohen’s kappa analysis was used to determine the inter-observer and intra-observer agreement of the fix-and-follow grading system. Values were interpreted using the following criteria: 0–0.20, poor; 0.21–0.40, fair; 0.41–0.60, moderate; 0.61–0.80, good; and 0.81–1.00, excellent [5]. All statistical analyses were performed using SPSS for Windows version 21.0 software (SPSS, Chicago, IL, USA) and *p* < 0.05 was deemed to indicate statistical significance.

## Results

Twenty-one patients were included in this study and clinical characteristics were described in Table 2. The mean age of the patients at the initial ophthalmic examination was 16.8 months (range 2–36 months). Nine (42.9%) had history of birth injury or developmental delay. Fifteen (71.4%) had strabismus (6 exotropia, 7 esotropia and 2 vertical strabismus), and 5 (23.8%) exhibited abnormal ocular movement (3 abduction limitation, 1 superior oblique muscle palsy and 1 oculomotor apraxia).

**Table 2:** Clinical characteristics of the patients

**Age (months, mean ± SD)**	16.81 ± 9.94
**Sex (male**: **female)**	9:12
**History of birth injury or developmental delay (n. of the patients, (%))**	9 (42.9)
**Strabismus (n. of the patients, (%))**	15 (71.4)
**Exotropia**	6
**Esotropia**	7
**Vertical strabismus**	2
**Abnormal ocular movement (n. of the patients, (%))**	5 (23.8)
**Abduction limitation**	3
**Superior oblique palsy**	1
**Oculomotor apraxia**	1

Cross tabulation between two examinations in single observer **(**Table 3**)** and between two examiners **(**Table 4**)** was presented. Intra-observer agreement was excellent (Kappa coefficient; 0.823, *p* < 0.001) and inter-observer agreement was good (Kappa coefficient; 0.625, *p* < 0.001), respectively. There were no discrepancies exceeding 2 grades. In all 5 of the patients who exhibited abnormal ocular movements, there was a discrepancy between the scores assigned by the two examiners.

**Table 3:** Cross tabulation between two examinations in single observer

	Exam2						Total
	2	3	4	5	6	7	
**Exam1**							
**2**	**1**	0	0	0	0	0	1
**3**	0	**2**	0	0	0	0	2
**4**	0	0	**3**	0	0	0	3
**5**	0	0	0	**4**	2	0	6
**6**	0	0	0	0	**4**	0	4
**7**	0	0	0	0	1	**4**	5
**Total**	1	2	3	4	7	4	21

**Table 4:** Cross tabulation between two examiners

	Observer1						Total
	2	3	4	5	6	7	
**Observer2**							
**2**	**1**	0	0	0	0	0	1
**3**	0	**2**	0	0	0	0	2
**4**	0	0	**2**	0	0	0	2
**5**	0	0	1	**0**	0	0	1
**6**	0	0	0	4	**6**	0	10
**7**	0	0	0	0	1	**4**	5
**Total**	1	2	3	4	7	4	21

## Discussion

In our study, both interobserver and intraobserver agreement were below good in evaluating visual development via Fix-and-Follow scoring. Visual function is known to play a crucial role in early infantile development and assessing visual function can yield important information in children with development delay or neurologic disease. Several studies have investigated the assessment of visual function in infants [6–11]. Rossi et al. [12] proposed an assessment method called ‘NAVEG’ to investigate ophthalmic, motor and perceptual components of visual function in neonates for screening purposes. The motor parameters assessed included fixation, horizontal/vertical smooth pursuit and saccadic movement. They reported good results with regard to distinguishing neurologic abnormalities in the subjects that fit well with the goals of the research. Because of the large number of items the NAVEG contains however, it takes a long time to perform and it can be difficult to analyse the final results. In addition, Rossi et al. [12] did not analyse the test’s reliability or reproducibility. Ricci et al. [13] suggested a range of test items for assessing visual function. In that study, 8 of a total of 13 items were related to ocular movement including fixation and tracking. The degree of fixation was classified into three stages, absent, unstable and stable. Horizontal and vertical arc tracking was divided into four categories, absent, brief, incomplete and complete. Although they demonstrated high concordance, specific data pertaining to inter-observer and intra-observer reliability were not reported and no comparisons were made with other ophthalmic factors such as eye movement.

Among the various methods for assessing visual function, fix-and-follow is the easiest for general ophthalmologists to perform, and it is likely to be the one with which they are the most familiar. It can be evaluated without any tools, and because most new-borns can reportedly already fix on and follow a target 48 h after birth the method is appropriate for screening problematic infants [14]. Saccadic eye movement refers to rapid movement of the eyes that involves orienting the fovea toward an object a person wants to see, and fixation is the ability to maintain focus on an object after a saccadic jump due to visual stimuli. Stable fixation can be achieved via continuous corrective saccade. To follow a moving object, maintenance of fixation and pursuing the moving stimulus are needed [1]. Therefore, fix-and-follow may be a good parameter for the assessment of early visual development.

Attempts have been made to predict sensory visual function using the fix-and-follow method. Atilla et al. [15] assessed the reliability of visual screening with a ‘fix-follow-maintain’ (FFM) method for the early detection of amblyopia. They assessed the use of FFM to investigate fix-and-follow monocularly and maintaining fixation binocularly and reported that three separate components of fixation were assessed; quality and accuracy, location, and duration. They concluded that the FFM method was not sensitive or specific, so amblyopia treatment should not be initiated solely based on FFM testing. Nevertheless, because the FFM method and Snellen visual acuity were not examined simultaneously and they did not provide objective classification criteria it cannot be argued that their method reflects visual acuity linearly. Kothari et al. [16] used central, steady, maintained fixation (CSM) grading for predicting inter-eye visual acuity differences in patients with horizontal strabismus. The movement of the non-dominant eye was observed while the dominant eye was covered. They concluded that CSM grading was useful for detecting the direction of strabismic amblyopia but not useful for quantitative evaluation. Notably, in the present study we did not investigate visual acuity as it was investigated in the studies described above. Instead we aimed to develop a quantitative and reproducible grading system for application under binocular conditions that can be used as a tool to screen early visual function, which may include both visual and visuo-cognitive components.

Due to the development of medical technology, the survival rate of premature infant, children with congenital disabilities or birth defect has been increasing. There is a need for a reliable and objective standard for the evaluation of visual development both for the early detection of ophthalmic problems and for use as an index of infant development. The new fix-and-follow grading system described herein can be applied easily in an office setting and characterizes the wide range of fix-and-follow statuses. The system proved easy to use in preverbal patients, and it does not require any expensive instruments. Though it is not designed to screen for specific medical conditions associated with visual acuity, the system does utilise discrete criteria for the quantitative measurement of fix-and-follow status. Notably, repeated testing and careful interpretation should be used in patients with ocular motility disorders.

The small number of samples may be a limitation of our study. Nevertheless, considering the high agreement shown in this study and that it is an evaluation tool that can be easily implemented without special tools, this limitation may be overcome through future studies with larger number of subjects. It was difficult to assess which factors influenced the agreements. Because the method does not explicitly distinguish between visual impairment and cognitive deficit, accompanying evaluation of cognitive or motor function may be needed in children with developmental disabilities. All 5 patients who had abnormal ocular motor disorders were associated with grading discrepancies between the examiners. Therefore, evaluating ocular motility disorders before assessing fix-and-follow performance would be an informative step and reduce the probability of obtaining unclear results.

In summary, our new fix-and-follow grading system described exhibited high repeatability and reproducibility in infants and children, who often do not have the capacity to cooperate with an examiner. It could be useful for both screening and longitudinal follow-up of visual function in preverbal children and easily applied in pracitce. However, the grading and interpretation should be conducted carefully in patients with ocular motility disorders.

## Data Availability

All data was included in the manuscript.
